# A Missense Variant in *SCN8A* in Alpine Dachsbracke Dogs Affected by Spinocerebellar Ataxia

**DOI:** 10.3390/genes10050362

**Published:** 2019-05-10

**Authors:** Anna Letko, Elisabeth Dietschi, Marco Nieburg, Vidhya Jagannathan, Corinne Gurtner, Anna Oevermann, Cord Drögemüller

**Affiliations:** 1 Institute of Genetics, Vetsuisse Faculty, University of Bern, 3012 Bern, Switzerland; anna.letko@vetsuisse.unibe.ch (A.L.); elisabeth.dietschi@vetsuisse.unibe.ch (E.D.); vidhya.jagannathan@vetsuisse.unibe.ch (V.J.); 2 Tierarztpraxis Nieburg, 18546 Sassnitz, Germany; marco@vetmed-berlin.de; 3 Institute of Animal Pathology, Vetsuisse Faculty, University of Bern, 3012 Bern, Switzerland; corinne.gurtner@vetsuisse.unibe.ch; 4 Division of Neurological Sciences, Vetsuisse Faculty, University of Bern, 3012 Bern, Switzerland; anna.oevermann@vetsuisse.unibe.ch

**Keywords:** *Canis familiaris*, sodium channel, central nervous system, ataxia, Alpine Dachsbracke

## Abstract

Spinocerebellar ataxias is an umbrella term for clinically- and neuropathologically-heterogeneous early-onset hereditary neurodegenerative diseases affecting several dog breeds. The purpose of this study is to identify the causative genetic variant associated with ataxia, tremor, and loss of balance in Alpine Dachsbracke dogs. We investigated two related litters in which four cases were reported. Neuropathology of two dogs revealed spongy degeneration associated with axonal degeneration. Combined genetic linkage and autozygosity analyses in four cases and eight related controls showed one critical disease-associated interval on chromosomes 27. Private whole-genome sequence variants of one ataxia case against 600 unrelated controls revealed one protein-changing variant within the critical interval in the *SCN8A* gene (c.4898G>T; p.Gly1633Val). Perfect segregation with the phenotype was confirmed by genotyping >200 Alpine Dachsbracke dogs. *SCN8A* encodes a voltage-gated sodium channel and the missense variant was predicted deleterious by three different in silico prediction tools. Pathogenic variants in *SCN8A* were previously reported in humans with ataxia, pancerebellar atrophy, and cognitive disability. Furthermore, cerebellar ataxia syndrome in the ‘jolting’ mutant mice is caused by a missense variant in *Scn8a*. Therefore, we considered the *SCN8A*:c.4898G>T variant to be the most likely cause for recessively inherited spinocerebellar ataxia in Alpine Dachsbracke dogs.

## 1. Introduction

Hereditary ataxias in humans are described as a clinically- and genetically-heterogeneous group of neurodegenerative diseases usually associated with cerebellar degeneration. They are phenotypically characterized by features such as gait abnormalities, imbalance, and associated movement abnormalities including incoordination of eye and hand movements, visual loss, seizures, behavioral symptoms, and peripheral neuropathy [1]. Different modes of inheritance have been described in cerebellar ataxias, with autosomal dominant (usually adult-onset) and autosomal recessive (typically juvenile-onset) being the most prevalent groups. Causative variants in more than 50 genes, which have been previously reported [2,3], reflect the large amount of clinical heterogeneity. Several pathophysiological mechanisms have been implicated including impaired ion channels function, failure of protein homeostasis, defects in the DNA repair system, or mitochondrial dysfunction [4].

Similar to humans, cerebellar ataxias in dogs are neurodegenerative disorders with variable age of onset, neuropathology, and disease severity. Cerebellar dysfunction is considered an important cause of movement disorders in purebred dogs [5]. However, to date, the number of described genes harboring the underlying genetic variants is significantly lower in dogs than in humans (*VLDLR*, *SPTBN2*, *SNX14*, *SEL1L*, *RAB24*, *KCNJ10*, *ITPR1*, *GRM1*, *CAPN1*, *ATP1B2*, *ATG4D*). These have been implicated in autosomal recessive ataxias of several dog breeds (OMIA 001947, 002092, 002034, 001692, 001913, 002089, 002097, 000078, 001820, 002110, 001954). Currently, canine neurodegenerative disease classification is based mainly on clinicopathological features, which due to lack of consensus may potentially lead to confusion and misdiagnoses. Therefore, it is important to improve the understanding of the underlying genetic and molecular mechanisms of inherited ataxias [5]. In this study, we aimed to identify the causative genetic variant associated with cerebellar ataxia, tremor, and loss of balance in Alpine Dachsbracke dogs.

## 2. Materials and Methods

### 2.1. Ethics Statement

All animal experiments were performed according to the local regulations. All animals in this study were examined with the consent of their owners. Sample collection was approved by the Cantonal Committee for Animal Experiments (Canton of Bern; permit 75/16).

### 2.2. DNA Samples and Single Nucleotide Variant Genotyping

A total of 216 blood samples from Alpine Dachsbracke dogs were collected. These included animals from two litters in which puppies affected by ataxia were observed (one dam, two sires, four cases, and eight healthy littermates). Genomic DNA was isolated from EDTA blood samples using the Maxwell RSC Whole Blood DNA kit (Promega, Dübendorf, Switzerland). DNA from the 12 selected dogs (4 affected and 8 closely related dogs, Supplementary Table S1) were genotyped using Illumina CanineHD BeadChip array (Illumina, San Diego, CA, USA) by GeneSeek (Neogen, Lincoln, NE, USA) for 220,853 single nucleotide variant (SNV) markers. Quality control filtering steps, as well as parentage control, were carried out using PLINK v1.9 [6]. Markers with call rate <90% were excluded, all individuals had call rates >90%. The pruned dataset consisted of 12 individuals and 213,288 markers.

### 2.3. Pathology

Brain from two affected puppies (Supplementary Table S1) and an eye from one affected puppy were collected and fixed in 4% buffered formaldehyde solution, embedded in paraffin, and sectioned at 4 µm. Sections were stained with hematoxylin and eosin (HE) and examined by light microscopy. Furthermore, selected sections of brains were stained with Bielschowsky silver stain.

### 2.4. Linkage and Autozygosity Analyses

**Figure 1:**
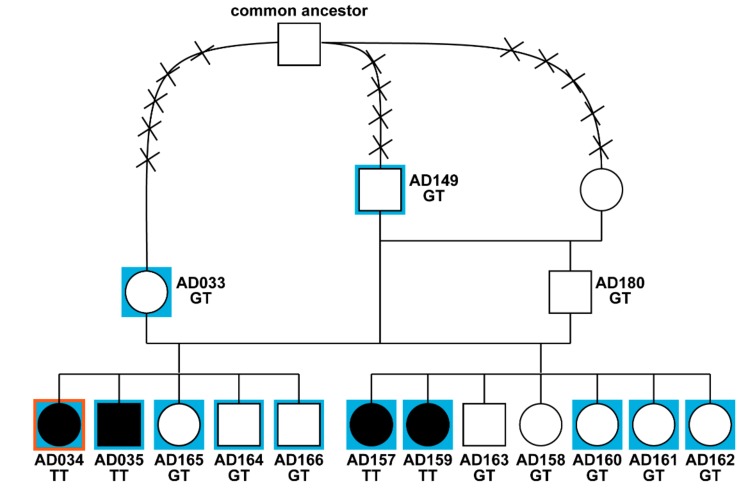
Pedigree of Alpine Dachsbracke dogs used for genetic mapping of spinocerebellar ataxia. Filled symbols represent spinocerebellar ataxia-affected dogs. The blue background indicates animals, which were genotyped on the single nucleotide variant (SNV) array, and the red contour indicates an affected dog selected for whole-genome resequencing. A common ancestor in both maternal and paternal lineages was identified. The crosses intersecting the connection lines represent the number of generations to the common ancestor. Note that the male ‘AD149’ sired the first litter and is also the grandsire of the second litter produced by his son ‘AD180’.

Linkage and autozygosity analyses were performed in order to find critical disease-associated intervals. Parametric linkage analysis was carried out under a fully penetrant, recessive model of inheritance using the Merlin software [7] to test for co-segregation of any chromosomal regions and the ataxia phenotype. The dataset for linkage analysis included one complete family of seven dogs representing the first litter (sire, dam, two affected and three unaffected offspring). Five additional littermates from the second litter (two affected and three unaffected) were added to the analysis carried out with PLINK v1.9 [6] to determine intervals of extended homozygous regions with alleles shared by all four affected dogs as well as individual homozygous intervals in the control animals (Figure 1). The circos plot was created using OmicCircos package [8].

### 2.5. Whole-Genome Resequencing

Whole-genome sequence (WGS) data at 23x coverage was obtained from a single affected Alpine Dachsbracke dog (Supplementary Table S1) after preparation of a PCR-free fragment library with a 450 bp insert size and collection of 206,013,034 paired-end reads (2 × 150 bp) using NovaSeq6000 Sequencing System (Illumina). The sequence data analysis and variant (SNVs and small indels) calling including the prediction of functional effects were performed as described before [9]. The dog reference genome assembly CanFam3.1 and NCBI annotation release 105 was used. The whole-genome sequence data from the Alpine Dachsbracke case was compared to the Boxer reference genome (CanFam3.1), 592 publically available control dogs of 124 various breeds, and 8 wolves (Supplementary Table S2). The Integrative genomics viewer (IGV) software [10] was used for visual inspection and screening for structural variants in the region of interest of the affected dog’s WGS.

### 2.6. Targeted Genotyping

Polymerase chain reaction (PCR) and Sanger sequencing were used to validate and genotype the variants identified from whole-genome resequencing. PCR products from genomic DNA were amplified using AmpliTaq Gold 360 Master Mix (Applied Biosystems, Foster City, CA, USA) and the purified PCR amplicons were directly sequenced on an ABI3730 capillary sequencer (Applied Biosystems). The *SCN8A* missense variant (XM_022411522.1:c.4898G>T) was genotyped using the following primers: GGCCAATGTTGAACAGAGCA (forward) and ACTTAAGGGCTCCAGTGTCA (reverse). The sequence data were analyzed using Sequencher 5.1 software (GeneCodes).

### 2.7. Protein Predictions

PON-P2 [11], PROVEAN [12], and MutPred2 [13] were used to predict biological consequences of the discovered variant on protein. All references to the canine *SCN8A* gene correspond to the accessions NC_006609.3 (NCBI accession), XM_022411522.1 (mRNA), and XP_022267230.1 (protein). The canine protein has the same length as the human SCN8A protein (NP_055006.1): 1980 amino acids from which 1966 (99.3%) are identical between dog and human. The Genome Aggregation Database (gnomAD) [14] was searched for corresponding variants in the human *SCN8A*.

### 2.8. Availability of Data and Material

The whole-genome data of an affected Alpine Dachsbracke dog has been made freely available (sample accession number SAMEA4848713) at the European Nucleotide Archive (ENA). All accession numbers of the control genomes are available in the Supplementary Table S2.

## 3. Results

### 3.1. Phenotype and Pedigree Analysis

We investigated two closely related litters of Alpine Dachsbracke dogs in which four puppies affected by ataxia were reported. All three parents of the affected dogs were phenotypically normal. A single female was sired with two related males (father and son) and pedigree analysis revealed a common ancestor within a maximum of five generations in both maternal and paternal lineages (Figure 1). Therefore, we assumed a monogenic autosomal recessive mode of inheritance.

Clinical signs of cerebellar dysfunction in the four puppies (one male, three females) were observed immediately when their normal littermates started to move in a coordinated fashion, so after approximately three weeks of age. The affected dogs exhibited ataxia, tremors, loss of balance, falling and other movement problems (Supplementary Video S1). Furthermore, the dog breeder reported that the vision of the affected dogs might be impaired. The severity of the clinical signs resulted in euthanasia of all cases by the age of 10–12 weeks.

Pedigree of Alpine Dachsbracke dogs used for genetic mapping of spinocerebellar ataxia.

### 3.2. Pathological Findings

**Figure 2:**
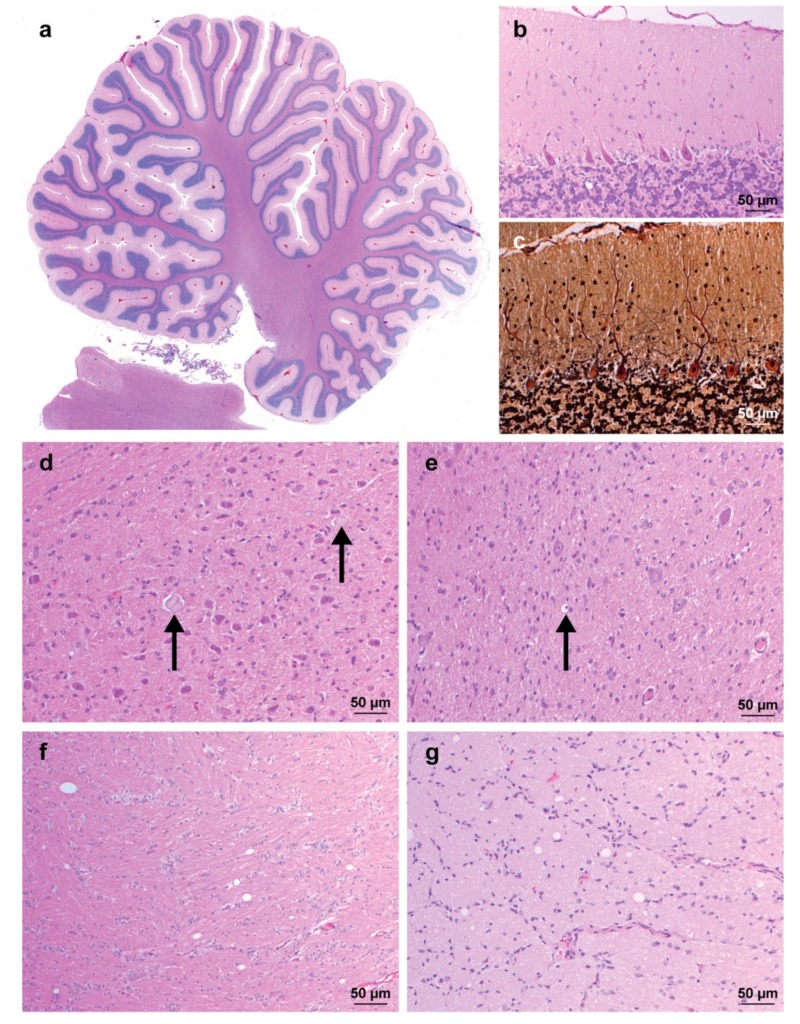
Neuropathology of a spinocerebellar ataxia-affected Alpine Dachsbracke dog. (**a**) Overview of the cerebellum showing a normal architecture. HE staining; (**b**) Cerebellar cortex with normal Purkinje cell layer. The molecular layer exhibits a slightly increased cellularity due to mild astrogliosis. Astrocytes have rather open-faced nuclei. Note the single, clearly delineated empty vacuole in the granule cell layer. Hematoxylin and eosin (HE) staining; (**c**) Cerebellar cortex with normal Purkinje cell layer. No empty baskets are observed. Bielschowsky stain; (**d**) Vestibulocochlear nucleus with marked astrogliosis and two axonal spheroids (arrows). HE staining; (**e**) Cerebellar nucleus showing marked astrogliosis and a single dilated myelin sheath with a central myelinophage (arrow). HE staining; (**f**) Corona radiata exhibiting diffuse astrogliosis and multiple clearly delineated empty vacuoles. HE staining; (**g**) Optic chiasm with multiple clearly delineated empty vacuoles. HE staining.

Neuropathological analysis revealed mild and scattered white and grey matter vacuolization indicating myelin splitting in the entire brain including the optic chiasm (Figure 2). The gross architecture of the cerebellum appeared normal, and no significant degeneration or loss of Purkinje cells and granule layer neurons was observed (Figure 2a). However, mild astrogliosis of the molecular layer and scattered vacuoles were present (Figure 2b). Additionally, mild to severe diffuse astrogliosis was observed in both white and grey matter, rarely associated with the presence of large axonal spheroids or axonal degeneration (Figure 2d,e). These changes were most severe in the vestibulocochlear nucleus, cerebellar nuclei, thalamus, and brainstem. In addition, the histopathological examination of the eye of one affected dog was carried out and showed no abnormalities. 

### 3.3. Positional Cloning of the Spinocerebellar Ataxia-Associated Locus

**Figure 3:**
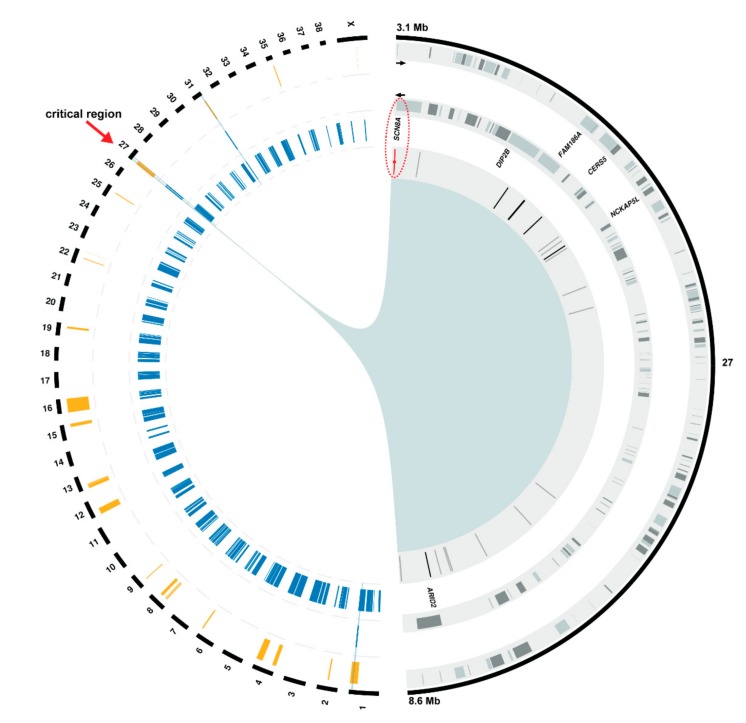
Positional cloning of the spinocerebellar ataxia-associated variant. The canine chromosomes are depicted in the left half of the circle as black bars. Below, the 3 circular tracks from outside to inside indicate: (i) 23 genome segments from linkage analysis in orange, (ii) four homozygous blocks shared in affected dogs in blue, (iii) homozygous blocks found in unaffected dogs in blue. The initial four segments overlapping in cases are indicated by a blue background. Only a single interval on chromosome 27 showed both linkage and homozygosity in affected dogs and no homozygosity in unaffected dogs. Therefore, it was considered the critical region for spinocerebellar ataxia (red arrow). The right half of the circle displays a close-up view of this region (chr27:3,154,712-8,679,881) with 3 circular tracks below showing from outside to inside: (i, ii) gene content showing the 165 genes (grey boxes) annotated on both, the positive (outer circle) and negative (inner circle) strand, (iii) location of private intergenic (grey), intronic (black), and exonic (red) SNVs depicted as vertical lines. For clarity, only the names of six genes, in which an intronic or exonic SNV was found, are shown. Note the red ellipse highlighting the single protein-changing SNV in the *SCN8A* gene.

Parametric linkage analysis for a recessive trait was performed in the complete litter and resulted in 23 linked genome regions with a positive logarithm of the odds (LOD) score (Supplementary Table S3). Based on the pedigree analysis we assumed that the affected dogs were inbred to a common founder and identical by descent (IBD) for the causative genetic variant and flanking chromosomal regions. Therefore, autozygosity mapping approach was used to determine regions of homozygosity shared across all four cases. Four genome regions with a total of ~22.4 Mb were identified (Supplementary Table S4). By overlapping the linked and homozygous intervals, four chromosomal segments (one on chromosomes 1, two on chromosome 27, and one on chromosome 31) remained as plausible regions of interest (Figure 3). Analyzing the homozygous regions in eight genotyped control dogs including six normal littermates and two parents revealed 144 blocks of homozygosity across the whole genome (Supplementary Table S5). We further looked for intervals showing both linkage and homozygosity in affected dogs and no homozygosity in any control dog. Only a single interval on chromosome 27, containing 269 SNVs and spanning ~5.5 Mb (chr27:3,154,712-8,679,881), remained as the most likely critical region for spinocerebellar ataxia (Figure 3).

### 3.4. Identification of the Causative Variant

In total, 165 genes were annotated in the disease-associated ~5.5 Mb critical interval on chromosome 27 (Figure 3). No structural variants were found by visual investigation of this genomic region in the whole-genome sequence of an affected dog. Filtering of the WGS data for private variants present in a homozygous state in the affected dog and absent in 600 control dog and wolf genomes yielded 21 SNVs within the critical interval (Supplementary Table S6). Six variants were located in introns of five genes (*DIP2B*, *FAM186A*, *CERS5*, *NCKAP5L*, and *ARID2*) while 14 others were intergenic. Only one exonic, protein-changing variant in a compelling functional candidate gene *SCN8A* (chr27:g.3,179,029C>A) was found (Figure 3).

**Figure 4:**
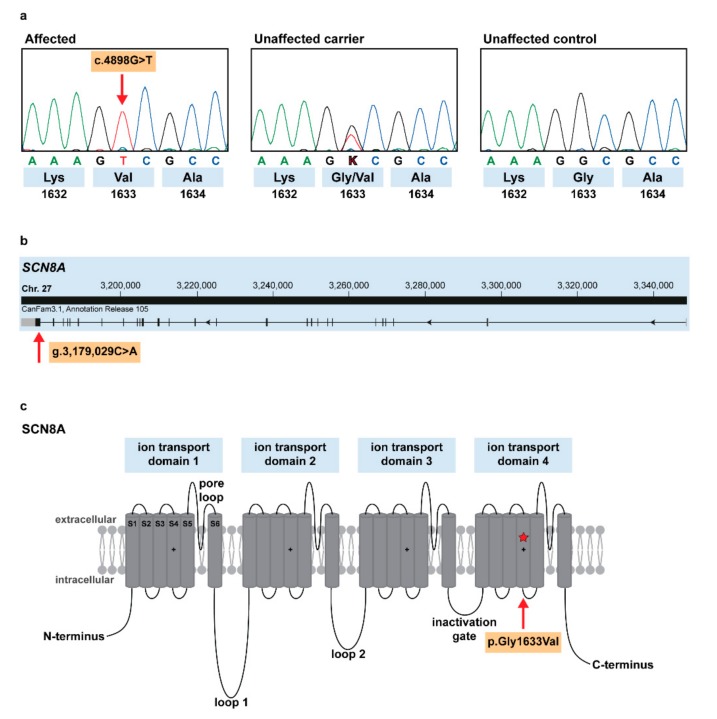
Spinocerebellar ataxia-associated SCN8A missense variant in Alpine Dachsbracke dogs. (**a**) Chromatograms of an affected, carrier, and wild type individual dogs, indicate the c.4898G>T variant, which changes codon 1633 (shown below); (**b**) *SCN8A* gene structure showing the variant location in exon 27 (red arrow); (**c**) Schematic representation of SCN8A protein and its four ion transport domains, which are separated by cytoplasmic loops and a short inactivation gate, each domain contains six transmembrane segments S1–S6 (adapted from [15]). Our p.Gly1633Val variant affects the positively charged S4 of the last domain (red star and arrow).

The detected variant (*SCN8A*:c.4898G>T) alters the encoded amino acid of SCN8A residue 1633 (p.Gly1633Val) with a predicted moderate impact on the resulting protein (Figure 4a). The missense variant is located in the last exon of the sodium voltage-gated channel α subunit 8 gene (Figure 4b) and affects the conserved ion transport domain 4 of SCN8A protein (Figure 4c). The α subunit consists of four homologous ion transport domains, each of which is organized in six transmembrane segments and contains a pore loop between the last two segments [15] (Figure 4c). The amino acid is highly conserved across species (Table S7) and the glycine to valine substitution was predicted pathogenic and deleterious by several in silico prediction tools (PON-P2 probability for pathogenicity: 0.891, MutPred2 score: 0.930, PROVEAN score: −7.256). Furthermore, there is no non-synonymous variant in the human *SCN8A* coding region at the corresponding position depicted in the gnomAD [14].

The perfect segregation of the detected *SCN8A* variant with the observed disease phenotype was further confirmed by genotyping all 216 available Alpine Dachsbracke dogs. Only the four affected dogs were homozygous for the variant allele, three obligate carriers, eight tested littermates in addition to eight controls were heterozygous carriers of the variant, whereas it was absent in 193 controls (Table 1).

## 4. Discussion

Based on clinicopathological data a novel congenital form of spinocerebellar ataxia was diagnosed in four Alpine Dachsbracke dogs. The neuropathological examination of two affected dogs indicated clear, even though not extremely severe, structural changes in the brain. Nevertheless, the observed pathological changes showed similarities to other spinocerebellar ataxias, such as *KCNJ10*-related spongy degeneration with cerebellar ataxia (SDCA1) in Belgian Shepherd Dogs (OMIA 002089) [9], and the vacuolization with marked astrogliosis indicated intramyelinic edema, which was compatible with an ion channel defect. We have used genome-wide SNV data for positional cloning of the disease-associated locus. Because of the population structure of the studied purebred Alpine Dachsbracke dogs, we were able to narrow down the locus by linkage analysis and autozygosity mapping to four chromosomal segments. Additionally, the normal related control dogs shared three of these homozygous intervals with identical alleles, which allowed us to focus on the ~5.5 Mb region on chromosome 27. Further molecular genetic analysis using current sequencing methods revealed a single most likely causative variant in a functional candidate gene.

*SCN8A* encodes the α subunit of a voltage-gated sodium channel Na_v_1.6, which is important for sodium ion transport to neurons in the central as well as peripheral nervous systems. However, it is most abundant in the maturing nodes of Ranvier in myelinated axons of the central nervous system, in which it contributes to nerve conduction velocity [16,17]. Na_v_1.6 was also found at the axon initial segment of excitatory and inhibitory neurons, where it regulates the formation of action potentials, which is essential for membrane depolarization [18]. Voltage-gated sodium channels consist of one α subunit forming the pore and up to two β subunits. The α subunit is organized in four repeated domains linked by cytoplasmic loops, and each domain is made up of six transmembrane segments (S1–S6). First four segments of each homologous domain form the primary voltage sensor for activation, and last two segments together with the pore loop form the ion pore. After depolarization, the positively charged S4 rotates and initiates a conformational change, which is important for opening the sodium channel pore. [16].

Altering of the sodium channels leads to abnormally, either increased or decreased, neuronal signaling as previously described in human patients suffering from epileptic encephalopathy [19]. Mutations of the Na_v_1.6 channel are described in ~1% of almost 1500 children affected by early-onset epileptic encephalopathies [20]. Protein-changing *SCN8A* variants have been previously associated with clinically similar movement disorders including cerebellar ataxia, pancerebellar atrophy, and cognitive disability in humans. Interestingly, all known *SCN8A*-associated human neurodegenerative diseases are caused by dominant acting variants (OMIM 600702).

The effects of loss of Na_v_1.6 channel have been extensively studied in spontaneous mouse mutants and can cause movement abnormalities, including ataxia, tremor, muscle weakness, and atrophy, or dystonia. Mice with null mutations exhibit hind limb paralysis, motor impairments, and early death, while heterozygous carriers show milder phenotypes [21]. A missense mutation in *Scn8a* is associated with cerebellar ataxia in the so-called ‘jolting’ mutant (Supplementary Table S7) and is a result of a shift in voltage dependence of the channel opening [22].

The herein described substitution in the Alpine Dachsbracke dogs alters a conserved position predicted to be in the transmembrane segment S4 in the fourth homologous domain; *in silico* analysis predicts the variant to be probably damaging to the protein structure/function. Heterozygous carriers do not exhibit a visible clinical phenotype, as they can most likely compensate due to the presence, albeit at a reduced level, of the normal proteins. As we identified a non-synonymous variant in a highly plausible functional candidate gene, our mapping analyses and whole-genome resequencing data combined with the current knowledge on SCN8A function in humans and mice strongly support the causality of *SCN8A*:c.4898G>T variant in the Alpine Dachsbracke breed. However, follow-up functional experiments would be needed to fully show the deleterious effects of the putative causal variant on the gene function.

In conclusion, our results provide strong evidence for a breed-specific missense variant in *SCN8A* gene as the most likely causative genetic variant for monogenic recessive spinocerebellar ataxia in Alpine Dachsbracke dogs. This is the first report of an *SCN8A*-associated form of ataxia in dogs. Our results enable the development of a genetic test for veterinary diagnostic and breeding purposes.

## Figures and Tables

**Table 1:** Segregation of the *SCN8A*:c.4898G>T genotypes with spinocerebellar ataxia in Alpine Dachsbracke dogs.

Disease Status	G/G	G/T	T/T
Affected (*n* = 4)	0	0	4
Non-affected (*n* = 212)	193	19 ^1^	0

^1^ includes 3 obligate carriers and 8 littermates of the affected dogs.

## Supplementary Materials

The following are available online at https://www.mdpi.com/2073-4425/10/5/362/s1, Table S1: Detailed information and genotypes of 216 Alpine Dachsbracke dogs, Table S2: Sample designations and breed information of all whole-genome sequences, Table S3: Genome regions with positive LOD scores for linkage to ataxia in the Alpine Dachsbracke family, Table S4: Shared homozygous regions across the four analyzed Alpine Dachsbracke ataxia cases, Table S5: Genome-wide homozygous regions in the eight analyzed control Alpine Dachsbracke dogs, Table S6: List of private variants of the sequenced Alpine Dachsbracke case in the region of interest on chromosome 27, Table S7: Alignment of SCN8A in vertebrate species. Video S1: Video illustrating the clinical phenotype of an affected Alpine Dachsbracke dog.
