# Supplementary material for: A Missense Variant in SCN8A in Alpine Dachsbracke Dogs Affected by Spinocerebellar Ataxia
Source: Genes (Basel). 2019 May 10;10(5):362. doi: 10.3390/genes10050362 (PMC6562999; doi:10.3390/genes10050362)
Supplement: Supplementary file 1 [file genes-10-00362-s001.zip › TableS7.pdf]

Table S7: Alignment of SCN8A in vertebrate species. The glycine residue that is substituted by a valine (p.Gly1633Val) in affected Alpine Dachsbracke dogs is highly conserved in the SCN8A orthologs (shown in yellow) similar to the 'jolting' mutant in mice (p.Ala1317Thr; Kohrmann et al. 1996) (shown in green).

Protein Acc.	Gene	Organism	Identity to *H. sapiens* (%)	
			Protein	DNA
NP_055006.1	SCN8A	*H.sapiens*	100	100
XP_001141985.3	SCN8A	*P.troglodytes*	99.5	99.2
XP_001090295.1	SCN8A	*M.mulatta*	99.9	98.8
XP_850134.1	SCN8A	*C.lupus*	99.4	95.4
NP_001180031.1	SCN8A	*B.taurus*	99.5	94.4
NP_001070967.1	Scn8a	*M.musculus*	98.7	90.1
NP_062139.2	Scn8a	*R.norvegicus*	98.7	90.7
XP_003643160.1	SCN8A	*G.gallus*	94.2	83.9
NP_001038648.1	scn8ab	*D.rerio*	83.6	76.3
NP_571703.1	scn8aa	*D.rerio*	84.3	76.0
XP_004911965.1	LOC101731347	*X.tropicalis*	88.1	77.1

```
NP_055006.1           1   MAARLLAPPGPDSFKPFTPESLANIERRIAESKLKKP--PKADGSHREDD   48
XP_001141985.3        1   MAARLLAPPGPDSFKPFTPESLANIERRIAESKLKKP--PKADGSHREDD   48
XP_001090295.1        1   MAARLLAPPGPDSFKPFTPESLANIERRIAESKLKKP--PKADGSHREDD   48
XP_850134.1           1   MAARLLAPPGPDSFKPFTPESLANIERRIAESKLKKP--PKAEGSHREDD   48
NP_001180031.1        1   MAARLLAPPGPDSFKPFTPESLANIERRIAESKLKKP--PKADGSHREDD   48
NP_001070967.1        1   MAARLLAPPGPDSFKPFTPESLANIERRIAESKLKKP--PKADGSHREDD   48
NP_062139.2           1   MAARLLAPPGPDSFKPFTPESLANIERRIAESKLKKP--PKADGSHREDD   48
XP_003643160.1        1   MAARLLAPPGPDSFKPFTPESLANIEKRIAEEKKKKR--PKQDSSHRDDD   48
NP_001038648.1        1   MAAPLLAPPGPDSFKRFTLESLANIEKRIAEEKKKKPVKPRSDSSHRDDD   50
NP_571703.1           1   MAAPLLAPPGPNSYKYFTRESLREIEKRIEEEKAKPP--PKPDNSYRDDD   48
XP_004911965.1            --------------------------------------------------

NP_055006.1          49   EDSKPKPNSDLEAGKSLPFIYGDIPQGLVAVPLEDFDPYYLTQKTFVVLN   98
XP_001141985.3       49   EDSKPKPNSDLEAGKSLPFIYGDIPQGLVAVPLEDFDPYYLTQKTFVVLN   98
XP_001090295.1       49   EDSKPKPNSDLEAGKSLPFIYGDIPQGLVAVPLEDFDPYYLTQKTFVVLN   98
XP_850134.1          49   EDSKPKPNSDLEAGKSLPFIYGDIPQGLVAVPLEDFDPYYLTQKTFVVLN   98
NP_001180031.1       49   EDSKPKPNSDLEAGKSLPFIYGDIPQGLVAVPLEDFDPYYLTQKTFVVLN   98
NP_001070967.1       49   EDSKPKPNSDLEAGKSLPFIYGDIPQGLVAVPLEDFDPYYLTQKTFVVLN   98
NP_062139.2          49   EDSKPKPNSDLEAGKSLPFIYGDIPQGLVAVPLEDFDPYYLTQKTFVVLN   98
XP_003643160.1       49   EDSKPKPNSDLEAGKNLPFIYGDIPKGLVAVPLEDFDPYYMTQKTFVVLN   98
NP_001038648.1       51   DDNKPKPNSDLEAGKGVPFIYGDVPSNMLAVPLEDLDPFYLNKKTFIVLN   100
NP_571703.1          49   DENKPKPNGDLEAGKSLPFIYGDIPPGMVATPLEDFDPFYINQKTFVVLN   98
XP_004911965.1            --------------------------------------------------

NP_055006.1          99   RGKTLFRFSATPALYILSPFNLIRRIAIKILIHSVFSMIIMCTILTNCVF   148
XP_001141985.3       99   RGKTLFRFSATPALYILSPFNLIRRIAIKILIHSVFSMIIMCTILTNCVF   148
XP_001090295.1       99   RGKTLFRFSATPALYILSPFNLIRRIAIKILIHSVFSMIIMCTILTNCVF   148
XP_850134.1          99   RGKTLFRFSATPALYILSPFNLIRRIAIKILIHSVFSMIIMCTILTNCVF   148
NP_001180031.1       99   RGKTLFRFSATPALYILSPFNLIRRIAIKILIHSVFSMIIMCTILTNCVF   148
NP_001070967.1       99   RGKTLFRFSATPALYILSPFNLIRRIAIKILIHSVFSMIIMCTILTNCVF   148
NP_062139.2          99   RGKTLFRFSATPALYILSPFNLIRRIAIKILIHSVFSMIIMCTILTNCVF   148
XP_003643160.1       99   RGKTLFRFSATPALYILSPFNLFRRIAIKILIHSVFSMIIMCTILTNCVF   148
NP_001038648.1      101   KGKTIFRFSATPSLYIISPFNLFRQIAIKILIHSFFSMIIMCTILTNCVF   150
NP_571703.1          99   KGKTIFRFSATPALYMISPFNLARRIAIKILIHSVFSMFIMCTILTNCVF   148
XP_004911965.1            --------------------------------------------------

NP_055006.1         149   MTFSNPPDWSKNVEYTFTGIYTFESLVKIIARGFCIDGFTFLRDPWNWLD   198
XP_001141985.3      149   MTFSNPPDWSKNVEYTFTGIYTFESLVKIIARGFCIDGFTFLRDPWNWLD   198
XP_001090295.1      149   MTFSNPPDWSKNVEYTFTGIYTFESLVKIIARGFCIDGFTFLRDPWNWLD   198
XP_850134.1         149   MTFSNPPEWSKNVEYTFTGIYTFESLVKIIARGFCIDGFTFLRDPWNWLD   198
NP_001180031.1      149   MTFSNPPDWSKNVEYTFTGIYTFESLVKIIARGFCIDGFTFLRDPWNWLD   198
NP_001070967.1      149   MTFSNPPEWSKNVEYTFTGIYTFESLVKIIARGFCIDGFTFLRDPWNWLD   198
NP_062139.2         149   MTFSNPPEWSKNVEYTFTGIYTFESLVKIIARGFCIDGFTFLRDPWNWLD   198
XP_003643160.1      149   MTFSNPPEWSKNVEYTFTGIYTFESLVKIIARGFCIDGFTFLRDPWNWLD   198
NP_001038648.1      151   MTFSNPPEWSKQVEYTFTGIYTFESLTKIVARGFCIDDFTFLRDPWNWLD   200
NP_571703.1         149   MTFSNPPEWSKQVEYTFTGIYTFESAVKIIARGFCIDGFTFLRDPWNWLD   198
XP_004911965.1            --------------------------------------------------
```

```
NP_055006.1        199   FSVIMMAYITEFVNLGNVSALRTFRVLRALKTISVIPGLKTIVGALIQSV   248
XP_001141985.3     199   FSVIMMAYVTEFVDLGNVSALRTFRVLRALKTISVIPGLKTIVGALIQSV   248
XP_001090295.1     199   FSVIMMAYITEFVNLGNVSALRTFRVLRALKTISVIPGLKTIVGALIQSV   248
XP_850134.1        199   FSVIMMAYITEFVNLGNVSALRTFRVLRALKTISVIPGLKTIVGALIQSV   248
NP_001180031.1     199   FSVIMMAYITEFVNLGNVSALRTFRVLRALKTISVIPGLKTIVGALIQSV   248
NP_001070967.1     199   FSVIMMAYITEFVNLGNVSALRTFRVLRALKTISVIPGLKTIVGALIQSV   248
NP_062139.2        199   FSVIMMAYVTEFVDLGNVSALRTFRVLRALKTISVIPGLKTIVGALIQSV   248
XP_003643160.1     199   FSVIMMAYVTEFVDLGNVSALRTFRVLRALKTISVIPGLKTIVGALIQSV   248
NP_001038648.1     201   FMVISMAYVTEFVDLGNVSALRTFRVLRALKTISVIPRLKTIVGALIQSV   250
NP_571703.1        199   FMVISMAYVTEFVDLGNVSALRTFRVLRALKTISVIPGLKTIVGALIQSV   248
XP_004911965.1           --------------------------------------------------

NP_055006.1        249   KKLSDVMILTVFCLSVFALIGLQLFMGNLRNKCVVWPINFNESYL-ENGT   297
XP_001141985.3     249   KKLSDVMILTVFCLSVFALIGLQLFMGNLRNKCVVWPINFNESYL-ENGT   297
XP_001090295.1     249   KKLSDVMILTVFCLSVFALIGLQLFMGNLRNKCVVWPINFNESYL-ENGT   297
XP_850134.1        249   KKLSDVMILTVFCLSVFALIGLQLFMGNLRNKCVVWPINFNESYL-ENGT   297
NP_001180031.1     249   KKLSDVMILTVFCLSVFALIGLQLFMGNLRNKCVVWPINFNESYL-ENGT   297
NP_001070967.1     249   KKLSDVMILTVFCLSVFALIGLQLFMGNLRNKCVVWPINFNESYL-ENGT   297
NP_062139.2        249   KKLSDVMILTVFCLSVFALIGLQLFMGNLRNKCVVWPINFNESYL-ENGT   297
XP_003643160.1     249   KKLSDVMILTVFCLSVFALIGLQLFMGNLRNKCVIWPINVNETYL-DNGS   297
NP_001038648.1     251   KKLSDVMILTVFCLSVFALIGLQLFMGNLRQKCVIWPVNSTETYL-ANGS   299
NP_571703.1        249   KKLSDVMILTVFCLSVFALIGLQLFMGNLRQKCVIWPINITEMFPDVNGS   298
XP_004911965.1           --------------------------------------------------

NP_055006.1        298   KGFDWEEYINNKTNFYTVPGMLEPLLCGNSSDAGQCPEGYQCMKAGRNPN   347
XP_001141985.3     298   KGFDWEEYINNKTNFYTVPGMLEPLLCGNSSDAGQCPEGYQCMKAGRNPN   347
XP_001090295.1     298   KGFDWEEYINNKTNFYTVPGMLEPLLCGNSSDAGQCPEGYQCMKAGRNPN   347
XP_850134.1        298   KGFDWEEYINNKTNFYIVPGMLEPLLCGNSSDAGQCPEGYQCMKAGRNPN   347
NP_001180031.1     298   KGFDWEEYINNKTNFYIVPGMLEPLLCGNSSDAGQCPEGYQCMKAGRNPN   347
NP_001070967.1     298   RGFDWEEYINNKTNFYMVPGMLEPLLCGNSSDAGQCPEGFQCMKAGRNPN   347
NP_062139.2        298   RGFDWEEYINNKTNFYMVPGMLEPLLCGNSSDAGQCPEGFQCMKAGRNPN   347
XP_003643160.1     298   RGFDWEEYTNNMSNFYIIPGAPDPLLCGNSSDAGQCPEGYTCMKAGRNPN   347
NP_001038648.1     300   KGFDFNEYIMNDTNFYFLPGMLDALLCGNSSDSGRCPEGYTCMKAGRNPN   349
NP_571703.1        299   RAFNWDEYILNETNFYFLPDQLDALLCGNSSDSGRCPEGYTCMKAGRNPN   348
XP_004911965.1           --------------------------------------------------

NP_055006.1        348   YGYTSFDTFSWAFLALFRLMTQDYWENLYQLTLRAAGKTYMIFFVLVIFV   397
XP_001141985.3     348   YGYTSFDTFSWAFLALFRLMTQDYWENLYQLTLRAAGKTYMIFFVLVIFV   397
XP_001090295.1     348   YGYTSFDTFSWAFLALFRLMTQDYWENLYQLTLRAAGKTYMIFFVLVIFV   397
XP_850134.1        348   YGYTSFDTFSWAFLALFRLMTQDYWENLYQLTLRAAGKTYMIFFVLVIFV   397
NP_001180031.1     348   YGYTSFDTFSWAFLALFRLMTQDYWENLYQLTLRAAGKTYMIFFVLVIFV   397
NP_001070967.1     348   YGYTSFDTFSWAFLALFRLMTQDYWENLYQLTLRAAGKTYMIFFVLVIFV   397
NP_062139.2        348   YGYTSFDTFSWAFLALFRLMTQDYWENLYQLTLRAAGKTYMIFFVLVIFV   397
XP_003643160.1     348   YGYTSFDTFSWAFLALFRLMTQDFWENLYQLTLRAAGKTYMIFFVLVIFV   397
NP_001038648.1     350   YGYTSFDSFGWAFLALFRLMTQDFWENLYQLTLRAAGKTYMIFFVLVIFV   399
NP_571703.1        349   YGYTSFDSFGWAFLALFRLMTQDFWENLYQLTLRAAGKTYMIFFVLVIFV   398
XP_004911965.1       1   ---------------------------------------MIFFVLVIFV   10

NP_055006.1        398   GSFYLVNLILAVVAMAYEEQNQATLEEAEQKEAEFKAMLEQLKKQQEEAQ   447
XP_001141985.3     398   GSFYLVNLILAVVAMAYEEQNQATLEEAEQKEAEFKAMLEQLKKQQEEAQ   447
XP_001090295.1     398   GSFYLVNLILAVVAMAYEEQNQATLEEAEQKEAEFKAMLEQLKKQQEEAQ   447
XP_850134.1        398   GSFYLVNLILAVVAMAYEEQNQATLEEAEQKEAEFKAMLEQLKKQQEEAQ   447
NP_001180031.1     398   GSFYLVNLILAVVAMAYEEQNQATLEEAEQKEAEFKAMLEQLKKQQEEAQ   447
NP_001070967.1     398   GSFYLVNLILAVVAMAYEEQNQATLEEAEQKEAEFKAMLEQLKKQQEEAQ   447
NP_062139.2        398   GSFYLVNLILAVVAMAYEEQNQATLEEAEQKEAEFKAMLEQLKKQQEEAQ   447
XP_003643160.1     398   GSFYLVNLILAVVAMAYEEQNQATLEEAEQKEAEFKAMLEQLKKQQEEAQ   447
NP_001038648.1     400   GSFYLVNLILAVVAMAYEEQNQATIEEAEQKEAEFKAMLEQLKKQQEEAQ   449
NP_571703.1        399   GSFYLVNLILAVVAMAYEEQNQATMEEAERKEAEFKAMLEQLKKQQEDAQ   448
XP_004911965.1      11   GSFYLVNLILAVVAMAYEEQNQATLEEAERKENEFKAMLEQMKRMQEEAQ   60

NP_055006.1        448   AAAMATSAGTVSEDAIEEEGEEGGGSPRSSSEISKLSSKSAKERRNRRKK   497
XP_001141985.3     448   AAAMATSAGTVSEDAIEEEGEEGGGSPRSSSEISKLSSKSAKERRNRRKK   497
XP_001090295.1     448   AAAMATSAGTVSEDAIEEEGEEGGGSPRSSSEISKLSSKSAKERRNRRKK   497
XP_850134.1        448   AAAMATSAGTVSEDAIEEEGEEGAGSPRSSSEISKLSSKSAKERRNRRKK   497
NP_001180031.1     448   AAAMATSAGTVSEDAIEEEGEDGAGSPRSSSEISKLSSKSAKERRNRRKK   497
NP_001070967.1     448   AAAMATSAGTVSEDAIEEEGEDGVGSPRSSSELSKLSSKSAKERRNRRKK   497
NP_062139.2        448   AAAMATSAGTVSEDAIEEEGEDGVGSPRSSSELSKLSSKSAKERRNRRKK   497
XP_003643160.1     448   AAAMVTSAGTVSEDAVEDDG--GGRMSRSSSEISKLSSKSAKERRNRRKK   495
NP_001038648.1     450   ANAMATSAGTVSEDAVEDDGG--GHLSRSSSEVSKLSSKSAKERRNRKKK   497
NP_571703.1        449   ANAMATSAGTVSEDVVEDDGDGEGNLSCSSSEMSKLSSKSAKERRNRKKK   498
XP_004911965.1      61   AAANATSAGTVTEDAVEDEDGE-ARMSHTSSEFSKPSSKSAKERRNRRKK   109

NP_055006.1        498   RKQKELSEGEEKGDPEKVFKSESEDGMRRKAFRLPDNRIGRKFSIMNQSL   547
XP_001141985.3     498   RKQKELSEGEEKGDPEKVFKSESEDGMRRKAFRLPDNRIGRKFSIMNQSL   547
XP_001090295.1     498   RKQKELSEGEEKGDPEKVFKSESEDGMRRKAFRLPDNRIGRKFSIMNQSL   547
XP_850134.1        498   RKQKELSEGEEKGDPEKVFKSESEDGMRRKGFRLPDNRIGRKFSIMNQSL   547
NP_001180031.1     498   RKQKELSEGEEKGDPEKVFKSESEDGMRRKGFRLPDNRIGRKFSIMNQSL   547
NP_001070967.1     498   RKQKELSEGEEKGDPEKVFKSESEDGMRRKAFRLPDNRIGRKFSIMNQSL   547
NP_062139.2        498   RKQKELSEGEEKGDPEKVFKSESEDGMRRKAFRLPDNRIGRKFSIMNQSL   547
XP_003643160.1     496   RKDKELSEGEEKGDNEKVFKSESEDGMRRKAFRLPDNRIGRKFSIMNQSL   545
NP_001038648.1     498   WRKEQ--E--EKGDSEKVVKSESDDGSKRSRFRFRDNRLGRKASIMNQSL   543
NP_571703.1        499   WRQKE--QDKEKGDSEKFVKSESDDGSRR-RFRFPDNRLGRRSSIMNQSL   545
XP_004911965.1     110   RKQKELSEGEEKGDSEKVYKSESEDGVKRRGFWYPDNRLGRRFSIMNQSL   159
```

```
NP_055006.1         548   LSIPGSPFLSRHNSKSSIFSFRGPGRFRDPGSENEFADDEHSTVEESEGR   597
XP_001141985.3      548   LSIPGSPFLSRHNSKSSIFSFRGPGRFRDPGSENEFADDEHSTVEESEGR   597
XP_001090295.1      548   LSIPGSPFLSRHNSKSSIFSFRGPGRFRDPGSENEFADDEHSTVEESEGR   597
XP_850134.1         548   LSIPGSPFLSRHNSKSSIFSFRGPGRFRDPGSENEFADDEHSTVEESEGR   597
NP_001180031.1      548   LSIPGSPFLSRHNSKSSIFSFRGPGRFRDPGSENEFADDEHSTVEESEGR   597
NP_001070967.1      548   LSIPGSPFLSRHNSKSSIFSFRGPGRFRDPGSENEFADDEHSTVEESEGR   597
NP_062139.2         548   LSIPGSPFLSRHNSKSSIFSFRGPGRFRDPGSENEFADDEHSTVEESEGR   597
XP_003643160.1      546   LSIPGSPFLSRHNSKSSIFSYK--GRFRDPGSENEFADDEHSTVEESEGR   593
NP_001038648.1      544   LSIPGSPFMSRR---SSIFSF--------KGSENEFADDEHSTVEESEDR   582
NP_571703.1         546   LSIPGSPFPSRRNSKSSIFSRC-----KDGGSENEFADDEHSTVEEYDER   590
XP_004911965.1      160   LSIPGSPYLSRHNSKSSIFSFR--GRFRDPVSENEFADDEHSTVEESEGR   207

NP_055006.1         598   RDSLFIPIRARERRSSYSGYSGYSQGSRSSRIFPSLRRSVKRNSTVDCNG   647
XP_001141985.3      598   RDSLFIPIRARERRSSYSGYSGYSQGSRSSRIFPSLRRSVKRNSTVDCNG   647
XP_001090295.1      598   RDSLFIPIRARERRSSYSGYSGYSQGSRSSRIFPSLRRSVKRNSTVDCNG   647
XP_850134.1         598   RDSLFIPIRARERRSSYSGYSGYSQGSRSSRIFPSLRRSVKRNSTVDCNG   647
NP_001180031.1      598   RDSLFIPIRARERRSSYSGYSGYSQGSRSSRIFPSLRRSVKRNSTVDCNG   647
NP_001070967.1      598   RDSLFIPIRARERRSSYSGYSGYSQCSRSSRIFPSLRRSVKRNSTVDCNG   647
NP_062139.2         598   RDSLFIPIRARERRSSYSGYSGYSQCSRSSRIFPSLRRSVKRNSTVDCNG   647
XP_003643160.1      594   RDSLFIPIRGRDRRSSYSGYSGYSQGSRSSRIFPNLRRNIKRNSTVDCNG   643
NP_001038648.1      583   RGSLFVP----YRRSSYSGYSQGSSRINPLAPHP----GCKRNSTVDCNG   624
NP_571703.1         591   RDSFLSP----QRRSSYTGFYG-----------------KRNSTVDCNG   618
XP_004911965.1      208   RDSLFIPVRGHDRRNSYN--SGYSQGSRSSRILQNLRRNPKRNSTVDCNG   255

NP_055006.1         648   VVSLIGGPGSHI-GGRLLPE-----------ATTEVEIKKKGPGSLLVSM   685
XP_001141985.3      648   VVSLIGGPGSHI-GGRLLPE-----------ATTEVEIKKKGPGSLLVSM   685
XP_001090295.1      648   VVSLIGGPGSHI-GGRLLPE-----------ATTEVEIRKKGPGSLLVSM   685
XP_850134.1         648   VVSLIGGPGSHI-GGRLLPE-----------ATTEVEIKKKGPGSLLVSM   685
NP_001180031.1      648   VVSLIGGPGSHI-SGRLLPE-----------ATTEVEIKKKGPGSLLVSM   685
NP_001070967.1      648   VVSLI-GPGSHI--GRLLPE-----------ATTEVEIKKKGPGSLLVSM   683
NP_062139.2         648   VVSLI-GPGSHI--GRLLPE-----------ATTEVEIKKKGPGSLLVSM   683
XP_003643160.1      644   VVSLIGGPPSSMPGGRLLPE-----------GTTEIEIKKKPGGSLLVSM   682
NP_001038648.1      625   VVSLI-GPG---P-GRLLPE------------TTEVEGKRKHSGSLMVSV   657
NP_571703.1         619   VVSLI-GPG---PGGRLLPE-----------PTTDLEIKKKLSGSLMVSV   653
XP_004911965.1      256   VVSLIGGPGSNIPGGRLLPEVKIDKAATDDSPTTEVEVKRKPSGSLRVSM   305

NP_055006.1         686   DQL-ASYGRKDRINSIMSVVTNTLVEELEESQRKCPPCWYKFANTFLIWE   734
XP_001141985.3      686   DQL-ASYGRKDRINSIMSVVTNTLVEELEESQRKCPPCWYKFANTFLIWE   734
XP_001090295.1      686   DQL-ASYGRKDRINSIMSVVTNTLVEELEESQRKCPPCWYKFANTFLIWE   734
XP_850134.1         686   DQL-ASYGRKDRINSILSVVTNTLVEELEESQRKCPPCWYKFANTFLIWE   734
NP_001180031.1      686   DQL-ASYGRKDRINSILSVVTNTLVEELEESQRKCPPCWYKFANTFLIWE   734
NP_001070967.1      684   EQL-ASYGRKDRINSIMSVVTNTLVEELEESQRKCPPCWYKFANTFLIWE   732
NP_062139.2         684   DQL-ASYGRKDRINSIMSVVTNTLVEELEESQRKCPPCWYKFANTFLIWE   732
XP_003643160.1      683   DQVNASYGRKDRTNSVMTGLTNTLVEELEESQRKCPPCWYKFANTFLIWE   732
NP_001038648.1      658   DQLNTSFSRKERANSAMTVVTNTLVEELEESQRKCPPCWYKFANTFLIWE   707
NP_571703.1         654   EQLNTSFGRKERANSVMSALTNTLVEELEESQRKCPPCWYKFANTFLIWE   703
XP_004911965.1      306   DQLTASFGRK-RANSILSVVTQTLVEELEESQRKCPPCWYKFADTYLIWT   354

NP_055006.1         735   CHPYWIKLKEIVNLIVMDPFVDLAITICIVLNTLFMAMEHHPMTPQFEHV   784
XP_001141985.3      735   CHPYWIKLKEIVNLIVMDPFVDLAITICIVLNTLFMAMEHHPMTPQFEHV   784
XP_001090295.1      735   CHPYWIKLKEIVNLIVMDPFVDLAITICIVLNTLFMAMEHHPMTPQFEHV   784
XP_850134.1         735   CHPYWIKLKEIVNLIVMDPFVDLAITICIVLNTLFMAMEHHPMTPQFEHV   784
NP_001180031.1      735   CHPYWIKLKEIVNLIVMDPFVDLAITICIVLNTLFMAMEHHPMTPQFEHV   784
NP_001070967.1      733   CHPYWIKLKEIVNLIVMDPFVDLAITICIVLNTLFMAMEHHPMTPQFEHV   782
NP_062139.2         733   CHPYWIKLKEIVNLIVMDPFVDLAITICIVLNTLFMAMEHHPMTPQFEHV   782
XP_003643160.1      733   CHPYWIKLKEIVNLIVMDPFVDLAITICIVLNTLFMAMEHHPMTPEFEHV   782
NP_001038648.1      708   CCPIWIKIKEIVNLIVMDPFVDLAITICIVLNTVFMAMEHYPMTPRFEEV   757
NP_571703.1         704   CGPLWMSIKEIVNLIVMDPFVDLAITICIVLNTLFMAMEHYPMTPQFEHV   753
XP_004911965.1      355   CCPLWIRIKTIVNMIVMDPFVDLTITICIVLNTLFMAMEHYPMTPHFENV   404

NP_055006.1         785   LAVGNLVFTGIFTAEMFLKLIAMDPYYYFQEGWNIFDGFIVSLSLMELSL   834
XP_001141985.3      785   LAVGNLVFTGIFTAEMFLKLIAMDPYYYFQEGWNIFDGFIVSLSLMELSL   834
XP_001090295.1      785   LAVGNLVFTGIFTAEMFLKLIAMDPYYYFQEGWNIFDGFIVSLSLMELSL   834
XP_850134.1         785   LAVGNLVFTGIFTAEMFLKLIAMDPYYYFQEGWNIFDGFIVSLSLMELGL   834
NP_001180031.1      785   LAVGNLVFTGIFTAEMFLKLIAMDPYYYFQEGWNIFDGFIVSLSLMELSL   834
NP_001070967.1      783   LAVGNLVFTGIFTAEMFLKLIAMDPYYYFQEGWNIFDGFIVSLSLMELGL   832
NP_062139.2         783   LAVGNLVFTGIFTAEMFLKLIAMDPYYYFQEGWNIFDGFIVSLSLMELSL   832
XP_003643160.1      783   LSVGNLVFTGIFTAEMFLKLIAMDPYYYFQEGWNIFDGFIVSLSLMELSL   832
NP_001038648.1      758   LSVGNLVFTGIFTAEMFAKLIAMDPYYYFQEGWNIFDGFIVSLSLVELGL   807
NP_571703.1         754   LSVGNLVFTGIFTAEMFAKLVAMDPYYYFQEGWNIFDGFIVSLSLMELGL   803
XP_004911965.1      405   LVVGNLVFTGIFTAEMFLKLIAMDPYYYFQEGWNIFDGFIVSLSLMELGL   454

NP_055006.1         835   ADVEGLSVLRSFRLLRVFKLAKSWPTLNMLIKIIGNSVGALGNLTLVLAI   884
XP_001141985.3      835   ADVEGLSVLRSFRLLRVFKLAKSWPTLNMLIKIIGNSVGALGNLTLVLAI   884
XP_001090295.1      835   ADVEGLSVLRSFRLLRVFKLAKSWPTLNMLIKIIGNSVGALGNLTLVLAI   884
XP_850134.1         835   ADVEGLSVLRSFRLLRVFKLAKSWPTLNMLIKIIGNSVGALGNLTLVLAI   884
NP_001180031.1      835   ADVEGLSVLRSFRLLRVFKLAKSWPTLNMLIKIIGNSVGALGNLTLVLAI   884
NP_001070967.1      833   ADVEGLSVLRSFRLLRVFKLAKSWPTLNMLIKIIGNSVGALGNLTLVLAI   882
NP_062139.2         833   ADVEGLSVLRSFRLLRVFKLAKSWPTLNMLIKIIGNSVGALGNLTLVLAI   882
XP_003643160.1      833   ANVEGLSVLRSFRLLRVFKLAKSWPTLNMLIKIIGNSVGALGNLTLVLAI   882
NP_001038648.1      808   ADVEGLSVLRSFRLLRVFKLAKSWPTLNMLIKIIGNSVGALGNLTLVLAI   857
NP_571703.1         804   ANVEGLSVLRSFRLLRVFKLAKSWPTLNMLIKIIGNSVGALGNLTLVLAI   853
XP_004911965.1      455   QDVEGLSVLRSFRLLRVFKLAKSWPTLNMLIKIIGNSVGALGNLTLVLAI   504
```

```
NP_055006.1        885   IVFIFAVVGMQLFGKSYKECVCKINQDCELPRWHMHDFFHSFLIVFRVLC   934
XP_001141985.3     885   IVFIFAVVGMQLFGKSYKECVCKINQDCELPRWHMHDFFHSFLIVFRVLC   934
XP_001090295.1     885   IVFIFAVVGMQLFGKSYKECVCKINQDCELPRWHMHDFFHSFLIVFRVLC   934
XP_850134.1        885   IVFIFAVVGMQLFGKSYKECVCKINQDCELPRWHMNDFFHSFLIVFRVLC   934
NP_001180031.1     885   IVFIFAVVGMQLFGKSYKECVCKINQDCELPRWHMHDFFHSFLIVFRVLC   934
NP_001070967.1     883   IVFIFAVVGMQLFGKSYKECVCKISQECKLPRWHMNDFFHSFLIVFRVLC   932
NP_062139.2        883   IVFIFAVVGMQLFGKSYKECVCKINQECKLPRWHMNDFFHSFLIVFRVLC   932
XP_003643160.1     883   IVFIFAVVGMQLFGKNYKECVCKINPECELPRWHMHDFFHSFLIVFRVLC   932
NP_001038648.1     858   IVFIFAVVGMQLFGKSYKDCVCKIAESCELPRWHMNDFFHSFLIVFRVLC   907
NP_571703.1        854   IVFIFAVVGMQLFGKSYKDCVCKIAQDCELPRWHMNDFFHSFLIVFRVLC   903
XP_004911965.1     505   IVFIFAVVGMQLFGKSYKDCVCKINPECVLPRWHMNDFFHSFLIVFRVLC   554

NP_055006.1        935   GEWIETMWDCMEVAGQAMCLIVFMMVMVIGNLVVLNLFLALLLSSFSADN   984
XP_001141985.3     935   GEWIETMWDCMEVAGQAMCLIVFMMVMVIGNLVVLNLFLALLLSSFSADN   984
XP_001090295.1     935   GEWIETMWDCMEVAGQAMCLIVFMMVMVIGNLVVLNLFLALLLSSFSADN   984
XP_850134.1        935   GEWIETMWDCMEVAGQAMCLIVFMMVMVIGNLVVLNLFLALLLSSFSADN   984
NP_001180031.1     935   GEWIETMWDCMEVAGQAMCLIVFMMVMVIGNLVVLNLFLALLLSSFSADN   984
NP_001070967.1     933   GEWIETMWDCMEVAGQAMCLIVFMMVMVIGNLVVLNLFLALLLSSFSADN   982
NP_062139.2        933   GEWIETMWDCMEVAGQAMCLIVFMMVMVIGNLVVLNLFLALLLSSFSADN   982
XP_003643160.1     933   GEWIETMWDCMEVAGQAMCLIVFMMVMVIGNLVVLNLFLALLLSSFSADN   982
NP_001038648.1     908   GEWIETMWDCMEVAGQSMCLIVFMMVMVIGNLVVLNLFLALLLSSFSADN   957
NP_571703.1        904   GEWIETMWDCMEVAGQAMCLIVFMMVMVIGNLVVLNLFLALLLSSFSADN   953
XP_004911965.1     555   GEWIETMWDCMEVAGQAMCIIVFMMVMVIGNLVVLNLFLALLLSSFSADN   604

NP_055006.1        985   LAATDDDGEMNNLQISVIRIKKGVAWTKLKVHAFMQAHFKQR-EADEVKP   1033
XP_001141985.3     985   LAATDDDGEMNNLQISVIRIKKGVAWTKLKVHAFMQAHFKQR-EADEVKP   1033
XP_001090295.1     985   LAATDDDGEMNNLQISVIRIKKGVAWTKLKVHAFMQAHFKQR-EADEVKP   1033
XP_850134.1        985   LAATDDDGEMNNLQISVIRIKKGVAWAKLKVHAFMQAHLKQR-EADEVKP   1033
NP_001180031.1     985   LAATDDDGEMNNLQISVIRIKKGVAWIKLKVHAFMQAHFKQR-EADEVKP   1033
NP_001070967.1     983   LAATDDDGEMNNLQISVIRIKKGVAWAKVKVHAFMQAHFKQR-EADEVKP   1031
NP_062139.2        983   LAATDDDGEMNNLQISVIRIKKGVAWTKVKVHAFMQAHFKQR-EADEVKP   1031
XP_003643160.1     983   LAATDDDGEMNNLQISVIRIKKGIAWTKAKVREFMQAHFKQR-EADEVKP   1031
NP_001038648.1     958   LAATDDDGEMNNLQISVIRIKKGIAWFKIHVRLLVAHVLKKKPLEDEDKP   1007
NP_571703.1        954   LAASDDDGEMNNLQIAVIRIKKGIAWVKAKVRELVNIILGRK-VTDEAKP   1002
XP_004911965.1     605   LAATDDDGEMNNLQISVIRIKKGIAWIKLKAHDFMQKHFKKK-DADEVKP   653

NP_055006.1       1034   LDELYEKKANCIANHTGADI-HRNGDFQKNGNGTTSGIGSSVEKYIIDED   1082
XP_001141985.3    1034   LDELYEKKANCIANHTGADI-HRNGDFQKNGNGTTSGIGSSVEKYIIDED   1082
XP_001090295.1    1034   LDELYEKKANCIANHTGADI-HRNGDFQKNGNGTTSGIGSSVEKYIIDED   1082
XP_850134.1       1034   LDELYEKKANCIANHTGADI-HRNGDFQKNGNGTTSGIGSSVEKYIIDED   1082
NP_001180031.1    1034   LDELYEKKANCIANHTGADI-HRNGDFQKNGNGTTSGIGSSVEKYIIDED   1082
NP_001070967.1    1032   LDELYEKKANCIANHTGVDI-HRNGDFQKNGNGTTSGIGSSVEKYIIDED   1080
NP_062139.2       1032   LDELYEKKANCIANHTGVDI-HRNGDFQKNGNGTTSGIGSSVEKYIIDED   1080
XP_003643160.1    1032   LDELYDKKVNCIANHTGADI-HRDIDYQKNGNGTTSGIGSSVEKYIIDED   1080
NP_001038648.1    1008   LDDMYDKKLNLMGNHTGVEIKCGDLNYPKNGNGTTSGIGSSVGKYMIDEE   1057
NP_571703.1       1003   LDDMYDRKLNCIANHTGVDI-SRDLDYQKNGNGTTSGIGSSVGKYMIDDD   1051
XP_004911965.1     654   LDEMYEKKMNCLANNTGADI-HRDMDYPKNGNGTTSGIGSSVEKYMIDED   702

NP_055006.1       1083   HMSFINNPNLTVRVPIAVGESDFENLNTEDVSSESDPEGSKDKLDDTSSS   1132
XP_001141985.3    1083   HMSFINNPNLTVRVPIAVGESDFENLNTEDVSSESDPEGSKDKLDDTSSS   1132
XP_001090295.1    1083   HMSFINNPNLTVRVPIAVGESDFENLNTEDVSSESDPEGSKDKLDDTSSS   1132
XP_850134.1       1083   HMSFINNPNLTVRVPIAVGESDFENLNTEDVSSESDPEGSKDKLDDTSSS   1132
NP_001180031.1    1083   HMSFINNPNLTVRVPIAVGESDFENLNTEDVSSESDPEGSKDKLDDTSSS   1132
NP_001070967.1    1081   HMSFINNPNLTVRVPIAVGESDFENLNTEDVSSESDPEGSKDKLDDTSSS   1130
NP_062139.2       1081   HMSFINNPNLTVRVPIAVGESDFENLNTEDVSSESDPEGSKDKLDDTSSS   1130
XP_003643160.1    1081   HMSFINNPNLTVRVPIAVGESDFENLNTEDFSSDTDPDGSKEKLDDTSSS   1130
NP_001038648.1    1058   PMSFIHNPNLTVRVPIAVGGSDFENLNTEDFSSESDVENSKD-LDDTSSS   1106
NP_571703.1       1052   HMSFIHNPNLTVCVPIAVGESDFENLNTEDFSSESEAEGSKE-LDDISSS   1100
XP_004911965.1     703   HMSFIHNPNLTVKVPIAVGESDFENLNTEDFSSASDEEGSKDKLDDTSSS   752

NP_055006.1       1133   EGSTIDIKPEVEE-VPVEQPEEYLDPDACFTEGCVQRFKCCQVNIEEGLG   1181
XP_001141985.3    1133   EGSTIDIKPEVEE-VPVEQPEEYLDPDACFTEGCVQRFKCCQVNIEEGLG   1181
XP_001090295.1    1133   EGSTIDIKPEVEE-VPVEQPEEYLDPDACFTEGCVQRFKCCQVNIEEGLG   1181
XP_850134.1       1133   EGSTIDIKPEVEE-VPVEQPEEYLDPDACFTEGCVQRFKCCQVNIEEGLG   1181
NP_001180031.1    1133   EGSTIDIKPEVEE-VPVEQPEEYLDPDACFTEGCVQRFKCCQVNIEEGLG   1181
NP_001070967.1    1131   EGSTIDIKPEVEE-VPVEQPEEYLDPDACFTEGCVQRFKCCQVNIEEGLG   1179
NP_062139.2       1131   EGSTIDIKPEVEE-VPVEQPEEYLDPDACFTEGCVQRFKCCQVNIEEGLG   1179
XP_003643160.1    1131   EGSTIDIKPEVEE-VPVEAPEEYLDPDACFTEGCMQRCKCCQVNIEEGLG   1179
NP_001038648.1    1107   EGSTIDIKPDEEEMAVVEVVEEYLDPEACWTDACVARYKCCDVPITEGWG   1156
NP_571703.1       1101   EGSTIDIKPEVEEAVVVETVEEYVDPEACWTEACIARYKCCDVPITEGWG   1150
XP_004911965.1     753   EGSTIDIKPEAEE-VPVEQPEEYLDPDNCFTEGCVARFKCCQVSVEEGLG   801

NP_055006.1       1182   KSWWILRKTCFLIVEHNWFETFIIFMILLSSGALAFEDIYIEQRKTIRTI   1231
XP_001141985.3    1182   KSWWILRKTCFLIVEHNWFETFIIFMILLSSGALAFEDIYIEQRKTIRTI   1231
XP_001090295.1    1182   KSWWILRKTCFLIVEHNWFETFIIFMILLSSGALAFEDIYIEQRKTIRTI   1231
XP_850134.1       1182   KSWWILRKTCFLIVEHNWFETFIIFMILLSSGALAFEDIYIEQRKTIRTI   1231
NP_001180031.1    1182   KSWWILRKTCFLIVEHNWFETFIIFMILLSSGALAFEDIYIEQRKTIRTI   1231
NP_001070967.1    1180   KSWWILRKTCFLIVEHNWFETFIIFMILLSSGALAFEDIYIEQRKTIRTI   1229
NP_062139.2       1180   KSWWILRKTCFLIVEHNWFETFIIFMILLSSGALAFEDIYIEQRKTIRTI   1229
XP_003643160.1    1180   KSWWTLRKTCFLIVEHNWFETFIIFMILLSSGALAFEDIYIEQRKTIRTI   1229
NP_001038648.1    1157   KYWWFLRKTCYLIVEHNWFETLIIFMILLSSGALAFEDVYIEQRKTIQII   1206
NP_571703.1       1151   KNWWFLRKTCYLIVEHNWFETLIIFMILLSSGALAFEDVYIEQRKTIRII   1200
XP_004911965.1     802   KSWWTLRKTCFLIVEHNWFETFIIFMILLSSGALAFEDVYIEQRKTIRTI   851
```

```
NP_055006.1       1232   LEYADKVFTYIFILEMLLKWTAYGFVKFFTNAWCWLDFLIVAVSLVSLIA   1281
XP_001141985.3    1232   LEYADKVFTYIFILEMLLKWTAYGFVKFFTNAWCWLDFLIVAVSLVSLIA   1281
XP_001090295.1    1232   LEYADKVFTYIFILEMLLKWTAYGFVKFFTNAWCWLDFLIVAVSLVSLIA   1281
XP_850134.1       1232   LEYADKVFTYIFILEMLLKWTAYGFVKFFTNAWCWLDFLIVAVSLVSLIA   1281
NP_001180031.1    1232   LEYADKVFTYIFILEMLLKWTAYGFVKFFTNAWCWLDFLIVAVSLVSLIA   1281
NP_001070967.1    1230   LEYADKVFTYIFILEMLLKWTAYGFVKFFTNAWCWLDFLIVAVSLVSLIA   1279
NP_062139.2       1230   LEYADKVFTYIFILEMLLKWTAYGFVKFFTNAWCWLDFLIVAVSLVSLIA   1279
XP_003643160.1    1230   LEYADKVFTYIFILEMLLKWCAYGFVKFFTNAWCWLDFLIVAVSLVSLIA   1279
NP_001038648.1    1207   LEYADRVFTYIFILEMLLKWVAYGFVKYFTNAWCWLDFFIVDVSIVSLIA   1256
NP_571703.1       1201   LEYADMVFTYIFILEMLLKWVAYGFVKYFTNAWCWLDFFIVDVSIVSLIA   1250
XP_004911965.1     852   LEYADKVFTYIFILEMLLKWLAYGFVKFFTNAWCWLDFLIVDVSLVSLIA   901

NP_055006.1       1282   NALGYSELGAIKSLRTLRALRPLRALSRFEGMRVVVNALVGAIPSIMNVL   1331
XP_001141985.3    1282   NALGYSELGAIKSLRTLRALRPLRALSRFEGMRVVVNALVGAIPSIMNVL   1331
XP_001090295.1    1282   NALGYSELGAIKSLRTLRALRPLRALSRFEGMRVVVNALVGAIPSIMNVL   1331
XP_850134.1       1282   NALGYSELGAIKSLRTLRALRPLRALSRFEGMRVVVNALVGAIPSIMNVL   1331
NP_001180031.1    1282   NALGYSELGAIKSLRTLRALRPLRALSRFEGMRVVVNALVGAIPSIMNVL   1331
NP_001070967.1    1280   NALGYSELGAIKSLRTLRALRPLRALSRFEGMRVVVNALVGAIPSIMNVL   1329
NP_062139.2       1280   NALGYSELGAIKSLRTLRALRPLRALSRFEGMRVVVNALVGAIPSIMNVL   1329
XP_003643160.1    1280   NALGYSELGAIKSLRTLRALRPLRALSRFEGMRVVVNALVGAIPSIMNVL   1329
NP_001038648.1    1257   NALGYSDLGPIKSLRTLRALRPLRALSRFEGMRVVVNALVGAIPSIMNVP   1306
NP_571703.1       1251   NALGYSDLGPIKSLRTLRALRPLRALSRFEGMRVVVNALVGAIPSIMNVL   1300
XP_004911965.1     902   NALGYSELGAIKSLRTLRALRPLRALSRFEGMRVVVNALVGAIPSIMNVL   951

NP_055006.1       1332   LVCLIFWLIFSIMGVNLFAGKYHYCFNETSEIRFEIEDVNNKTECEKLME   1381
XP_001141985.3    1332   LVCLIFWLIFSIMGVNLFAGKYHYCFNETSEIRFEIEDVNNKTECEKLME   1381
XP_001090295.1    1332   LVCLIFWLIFSIMGVNLFAGKYHYCFNETSEIRFEIEDVNNKTECEKLME   1381
XP_850134.1       1332   LVCLIFWLIFSIMGVNLFAGKYHYCFNETSEIRFDIEDVNNKTECEKLME   1381
NP_001180031.1    1332   LVCLIFWLIFSIMGVNLFAGKYHYCFNESSEIRFEIEDVNNKTECEKLME   1381
NP_001070967.1    1330   LVCLIFWLIFSIMGVNLFAGKYHYCFNETSEIRFEIDEVNNKTDCEKLME   1379
NP_062139.2       1330   LVCLIFWLIFSIMGVNLFAGKYHYCFNETSEIRFEIDIVNNKTDCEKLME   1379
XP_003643160.1    1330   LVCLIFWLIFSIMGVNLFAGKYHYCFNETSEERFEIEIVNNKTDCEALMP   1379
NP_001038648.1    1307   LVCLIFWLIFSIMGVNLFAGKYYYCFNQTAEEYFQVDVVNNKTECFALIN   1356
NP_571703.1       1301   LVCLIFWLIFSIMGVNMFAGKYYYCYNETEKAYFELDVVNNKSECFALIE   1350
XP_004911965.1     952   LVCLIFWLIFSIMGVNLFAGKYYYCYNETAEGMFEIEDVENETQCFDLIG   1001

NP_055006.1       1382   GNNTEIRWKNVKINFDNVGAGYLALLQVATFKGWMDIMYAAVDSRKPDEQ   1431
XP_001141985.3    1382   GNNTEIRWKNVKINFDNVGAGYLALLQVATFKGWMDIMYAAVDSRKPDEQ   1431
XP_001090295.1    1382   GNNTEIRWKNVKINFDNVGAGYLALLQVATFKGWMDIMYAAVDSRKPDEQ   1431
XP_850134.1       1382   GNNTEIRWKNVKINFDNVGAGYLALLQVATFKGWMDIMYAAVDSRKPDEQ   1431
NP_001180031.1    1382   GNNTEIRWKNVKINFDNVGAGYLALLQVATFKGWMDIMYAAVDSRKPDEQ   1431
NP_001070967.1    1380   GNNTEIRWKNVKINFDNVGAGYLALLQVATFKGWMDIMYAAVDSRKPDEQ   1429
NP_062139.2       1380   GNSTEIRWKNVKINFDNVGAGYLALLQVATFKGWMDIMYAAVDSRKPDEQ   1429
XP_003643160.1    1380   PNSTEIRWKNVKINFDNVGAGYLALLQVATFKGWMDIMYAAVDSRKQEEQ   1429
NP_001038648.1    1357   ANYTEVRWKNVKINFDNVGAGYLALLQVATFKGWMDIMYAAVDSRKVEDQ   1406
NP_571703.1       1351   QNYTEVRWKNVKINFDNVGAGYLALLQVATFKGWMDIMYAAVDSRRVEDQ   1400
XP_004911965.1    1002   NNYTEVRWKNVKINFDNVGAGYLALLQVATFKGWMDIMYAAVDSRKVHQQ   1051

NP_055006.1       1432   PKYEDNIYMYIYFVIFIIFGSFFTLNLFIGVIIDNFNQQKKKFGGQDIFM   1481
XP_001141985.3    1432   PKYEDNIYMYIYFVIFIIFGSFFTLNLFIGVIIDNFNQQKKKFGGQDIFM   1481
XP_001090295.1    1432   PKYEDNIYMYIYFVIFIIFGSFFTLNLFIGVIIDNFNQQKKKFGGQDIFM   1481
XP_850134.1       1432   PKYEDNIYMYIYFVIFIIFGSFFTLNLFIGVIIDNFNQQKKKFGGQDIFM   1481
NP_001180031.1    1432   PKYEDNIYMYIYFVIFIIFGSFFTLNLFIGVIIDNFNQQKKKFGGQDIFM   1481
NP_001070967.1    1430   PDYEGNIYMYIYFVIFIIFGSFFTLNLFIGVIIDNFNQQKKKFGGQDIFM   1479
NP_062139.2       1430   PDYEGNIYMYIYFVIFIIFGSFFTLNLFIGVIIDNFNQQKKKFGGQDIFM   1479
XP_003643160.1    1430   PKYEDNIYMYIYFVIFIIFGSFFTLNLFIGVIIDNFNQQKKKFGGQDIFM   1479
NP_001038648.1    1407   PLYEDNIYMYIYFVIFIIFGSFFTLNLFIGVIIDNFNQQKKKFGGQDIFM   1456
NP_571703.1       1401   PKYEDNIYMYIYFVIFIIFGSFFTLNLFIGVIIDNFNQQKKKFGGQDIFM   1450
XP_004911965.1    1052   PKYEDNIYMYIYFVIFIIFGSFFTLNLFIGVIIDNFNQQKKKFGGQDIFM   1101

NP_055006.1       1482   TEEQKKYYNAMKKLGSKKPQKPIPRPLNKIQGIVFDFVTQQAFDIVIMML   1531
XP_001141985.3    1482   TEEQKKYYNAMKKLGSKKPQKPIPRPL-----------------------   1508
XP_001090295.1    1482   TEEQKKYYNAMKKLGSKKPQKPIPRPLNKIQGIVFDFVTQQAFDIVIMML   1531
XP_850134.1       1482   TEEQKKYYNAMKKLGSKKPQKPIPRPLNKIQGIIFDFVTQQAFDIVIMML   1531
NP_001180031.1    1482   TEEQKKYYNAMKKLGSKKPQKPIPRPLNKIQGIVFDFVTQQAFDIVIMML   1531
NP_001070967.1    1480   TEEQKKYYNAMKKLGSKKPQKPIPRPLNKIQGIVFDFVTQQAFDIVIMML   1529
NP_062139.2       1480   TEEQKKYYNAMKKLGSKKPQKPIPRPLNKIQGIVFDFVTQQAFDIVIMML   1529
XP_003643160.1    1480   TEEQKKYYNAMKKLGSKKPQKPIPRPLNRIQGAVFDFVTQQAFDIVIMML   1529
NP_001038648.1    1457   PEEQKKYYNAMKKLGSKKPQKPIPRPLNKIQGMVFDFVTQQAFDISIMML   1506
NP_571703.1       1451   TEEQKKYYNAMKKLGSKKPQKPIPRPQNKLQGMVFDFVTQQVFDISIMIL   1500
XP_004911965.1    1102   TEEQKKYYNAMKKLGSKKPQKPIPRPLNKFQGAIFDIITQQAFDIVIMIL   1151

NP_055006.1       1532   ICLNMVTMMVETDTQSKQMENILYWINLVFVIFFTCECVLKMFALRHYYF   1581
XP_001141985.3    1509   -CL---------DGKRRERVSPISITXLLLFLFL----------------   1532
XP_001090295.1    1532   ICLNMVTMMVETDTQSKQMENILYWINLVFVIFFTCECVLKMFALRHYYF   1581
XP_850134.1       1532   ICLNMVTMMVETDTQSKQMENILYWINLVFVIFFTCECVLKMFALRHYYF   1581
NP_001180031.1    1532   ICLNMVTMMVETDTQSKQMENILYWINLVFVIFFTCECVLKMFALRHYYF   1581
NP_001070967.1    1530   ICLNMVTMMVETDTQSKQMENILYWINLVFVIFFTCECVLKMFALRHYYF   1579
NP_062139.2       1530   ICLNMVTMMVETDTQSKQMENILYWINLVFVIFFTCECVLKMFALRHYYF   1579
XP_003643160.1    1530   ICLNMVTMMVETDTQSKQMEDILYWINLVFVIFFTCECVLKMFALRHYYF   1579
NP_001038648.1    1507   ICLNMVTMMVETDDQSDETENILYWINFVFIVAFTGEFVLKLFALRHYYF   1556
NP_571703.1       1501   ICLNMVTMMVETDDQSQETENILYWINFIFIVAFTSEFVLKLFALRHYYF   1550
XP_004911965.1    1152   ICLNMVTMMVETDDQSDYTDNVLYWINVVFIVFFTTECVLKLCALRHYYF   1201
```

```
NP_055006.1       1582   TIGWNIFDFVVVILSIVGMFLADIIEKYFVSPTLFRVIRLARIGRILRLI   1631
XP_001141985.3    1533   -----------------GMFLADIIEKYFVSPTLFRVIRLARIGRILRLI   1565
XP_001090295.1    1582   TIGWNIFDFVVVILSIVGMFLADIIEKYFVSPTLFRVIRLARIGRILRLI   1631
XP_850134.1       1582   TIGWNIFDFVVVILSIVGMFLADIIEKYFVSPTLFRVIRLARIGRILRLI   1631
NP_001180031.1    1582   TIGWNIFDFVVVILSIVGMFLADIIEKYFVSPTLFRVIRLARIGRILRLI   1631
NP_001070967.1    1580   TIGWNIFDFVVVILSIVGMFLADIIEKYFVSPTLFRVIRLARIGRILRLI   1629
NP_062139.2       1580   TIGWNIFDFVVVILSIVGMFLADIIEKYFVSPTLFRVIRLARIGRILRLI   1629
XP_003643160.1    1580   TIGWNIFDFVVVILSIVGMFLAEIIEKYFVSPTLFRVIRLARIGRILRLI   1629
NP_001038648.1    1557   TNGWNVFDCVVVILSIVGMFLADLIEKYFVSPTLFRVIRLARIGRILRLI   1606
NP_571703.1       1551   TNGWNIFDCVVVILSIVGMFLADLIEKYFVSPTLFRVIRLARIGRILRLI   1600
XP_004911965.1    1202   TIGWNIFDFVVVILSIVGMFLADLITKYFVSPTLFRVIRLARIGRILRLI   1251

NP_055006.1       1632   KGAKGIRTLLFALMMSLPALFNIGLLLFLVMFIFSIFGMSNFAYVKHEAG   1681
XP_001141985.3    1566   KGAKGIRTLLFALMMSLPALFNIGLLLFLVMFIFSIFGMSNFAYVKHEAG   1615
XP_001090295.1    1632   KGAKGIRTLLFALMMSLPALFNIGLLLFLVMFIFSIFGMSNFAYVKHEAG   1681
XP_850134.1       1632   KGAKGIRTLLFALMMSLPALFNIGLLLFLVMFIFSIFGMSNFAYVKHEAG   1681
NP_001180031.1    1632   KGAKGIRTLLFALMMSLPALFNIGLLLFLVMFIFSIFGMSNFAYVKHEAG   1681
NP_001070967.1    1630   KGAKGIRTLLFALMMSLPALFNIGLLLFLVMFIFSIFGMSNFAYVKHEAG   1679
NP_062139.2       1630   KGAKGIRTLLFALMMSLPALFNIGLLLFLVMFIFSIFGMSNFAYVKHEAG   1679
XP_003643160.1    1630   KGAKGIRTLLFALMMSLPALFNIGLLLFLVMFIFSIFGMSNFAYVKHEAG   1679
NP_001038648.1    1607   KGAKGIRTLLFALMMSLPALFNIGLLLFLVMFIFSIFGMSNFAYVKREIG   1656
NP_571703.1       1601   KGAKGIRTLLFALMMSLPALFNIGLLLFLVMFIFSIFGMSNFAYVKRESG   1650
XP_004911965.1    1252   KGAKGIRTLLFALMMSLPALFNIGLLLFLVMFIFSIFGMSNFAYVKKEAG   1301

NP_055006.1       1682   IDDMFNFETFGNSMICLFQITTSAGWDGLLLPILNRPPDCSLDKEHPGSG   1731
XP_001141985.3    1616   IDDMFNFETFGNSMICLFQITTSAGWDGLLLPILNRPPDCSLDKEHPGSG   1665
XP_001090295.1    1682   IDDMFNFETFGNSMICLFQITTSAGWDGLLLPILNRPPDCSLDKEHPGSG   1731
XP_850134.1       1682   IDDMFNFETFGNSMICLFQITTSAGWDGLLLPILNRPPDCSLDKEHPGSG   1731
NP_001180031.1    1682   IDDMFNFETFGNSMICLFQITTSAGWDGLLLPILNRPPDCNLDKEHPGSG   1731
NP_001070967.1    1680   IDDMFNFETFGNSMICLFQITTSAGWDGLLLPILNRPPDCSLDKEHPGSG   1729
NP_062139.2       1680   IDDMFNFETFGNSMICLFQITTSAGWDGLLLPILNRPPDCSLDKEHPGSG   1729
XP_003643160.1    1680   IDDMFNFETFGNSMICLFQITTSAGWDGLLLPILNRPPDCDLDKEHPGSG   1729
NP_001038648.1    1657   IDDMYNFETFGNSMICLFMITTSAGWDGLLAPILNYPPDCDPNKENPGTT   1706
NP_571703.1       1651   IDDMYNFETFGNSMICLFMITTSAGWDGLLAPILNYPPDCEPTKENPGTS   1700
XP_004911965.1    1302   IDDMFNFETFGNSMICLFQITTSAGWDGLLLPILNRPPDCDAHKENPGSQ   1351

NP_055006.1       1732   FKGDCGNPSVGIFFFVSYIIISFLIVVNMYIAIILENFSVATEESADPLS   1781
XP_001141985.3    1666   FKGDCGNPSVGIFFFVSYIIISFLIVVNMYIAIILENFSVATEESADPLS   1715
XP_001090295.1    1732   FKGDCGNPSVGIFFFVSYIIISFLIVVNMYIAIILENFSVATEESADPLS   1781
XP_850134.1       1732   FKGDCGNPSVGIFFFVSYIIISFLIVVNMYIAIILENFSVATEESADPLS   1781
NP_001180031.1    1732   FKGDCGNPSVGIFFFVSYIIISFLIVVNMYIAIILENFSVATEESADPLS   1781
NP_001070967.1    1730   FKGDCGNPSVGIFFFVSYIIISFLIVVNMYIAIILENFSVATEESADPLS   1779
NP_062139.2       1730   FKGDCGNPSVGIFFFVSYIIISFLIVVNMYIAIILENFSVATEESADPLS   1779
XP_003643160.1    1730   FKGDCGNPSVGIFFFVSYIIISFLIVVNMYIAIILENFSVATEESADPLS   1779
NP_001038648.1    1707   VKGNCGNPSVGIFFFVMYIIVSFLIVVNMYIAIILENFSVATEESADPLC   1756
NP_571703.1       1701   VKGNCGNPSVGIFFFVMYIIVSFLIVVNMYIAIILENFSVATEESADPLC   1750
XP_004911965.1    1352   VKGDCGNPSVGIFFFVSYIIISFLIVVNMYIAIILENFSVATEESADPLS   1401

NP_055006.1       1782   EDDFETFYEIWEKFDPDATQFIEYCKLADFADALEHPLRVPKPNTIELIA   1831
XP_001141985.3    1716   EDDFETFYEIWEKFDPDATQFIEYCKLADFADALEHPLRVPKPNTIELIA   1765
XP_001090295.1    1782   EDDFETFYEIWEKFDPDATQFIEYCKLADFADALEHPLRVPKPNTIELIA   1831
XP_850134.1       1782   EDDFETFYEIWEKFDPDATQFIEYCKLADFADALEHPLRVPKPNTIELIA   1831
NP_001180031.1    1782   EDDFETFYEIWEKFDPDATQFIEYCKLADFADALEHPLRVPKPNTIELIA   1831
NP_001070967.1    1780   EDDFETFYEIWEKFDPDATQFIEYCKLADFADALEHPLRVPKPNTIELIA   1829
NP_062139.2       1780   EDDFETFYEIWEKFDPDATQFIEYCKLADFADALEHPLRVPKPNTIELIA   1829
XP_003643160.1    1780   EDDFETFYEIWEKFDPDATQFIEYSKLADFADALEHPLRVPKPNTIELIA   1829
NP_001038648.1    1757   EDDFESFYEIWEKFDPDASQFITFAKLPDFADTLEHPLRVPKPNIIELIA   1806
NP_571703.1       1751   EDDFESFYEIWEKFDPTASQFITFAKLGDFADTLEHPLRVPKPNTIELIA   1800
XP_004911965.1    1402   EDDFETFYEIWEKFDPDATQFIQFSKLPDFADALEHPLRIPKPNTIELIA   1451

NP_055006.1       1832   MDLPMVSGDRIHCLDILFAFTKRVLGDSGELDILRQQMEERFVASNPSKV   1881
XP_001141985.3    1766   MDLPMVSGDRIHCLDILFAFTKRVLGDSGELDILRQQMEERFVASNPSKV   1815
XP_001090295.1    1832   MDLPMVSGDRIHCLDILFAFTKRVLGDSGELDILRQQMEERFVASNPSKV   1881
XP_850134.1       1832   MDLPMVSGDRIHCLDILFAFTKRVLGDSGELDILRQQMEERFVASNPSKV   1881
NP_001180031.1    1832   MDLPMVSGDRIHCLDILFAFTKRVLGDSGELDILRQQMEERFVASNPSKV   1881
NP_001070967.1    1830   MDLPMVSGDRIHCLDILFAFTKRVLGDSGELDILRQQMEERFVASNPSKV   1879
NP_062139.2       1830   MDLPMVSGDRIHCLDILFAFTKRVLGDSGELDILRQQMEERFVASNPSKV   1879
XP_003643160.1    1830   MDLPMVSGDRIHCLDILFAFTKRVLGDSGELDILRQQMEERFVASNPSKV   1879
NP_001038648.1    1807   MDLPMVSGDRIHCLDILFAFTKRVLGDSGDLDMMRQQMEERFVAANPSKV   1856
NP_571703.1       1801   MDLPMVSGDRIHCLDILFAFTKRVLGDSGDLDMMRQQMEERFIAANPSKV   1850
XP_004911965.1    1452   MDLPMVSGDRIHCLDILFAFTKRVLGESGELDILRQQMEERFVASNPSKV   1501

NP_055006.1       1882   SYEPITTTLRRKQEEVSAVVLQRAYRGHLARRGFICKK-TTSNKLENGGT   1930
XP_001141985.3    1816   SYEPITTTLRRKQEEVSAVVLQRAYRGHLARRGFICKK-TTSNKLENGGT   1864
XP_001090295.1    1882   SYEPITTTLRRKQEEVSAVVLQRAYRGHLARRGFICKK-TTSNKLENGGT   1930
XP_850134.1       1882   SYEPITTTLRRKQEEVSAVVLQRAYRGHLARRGFICKK-TTSNKLENGGT   1930
NP_001180031.1    1882   SYEPITTTLRRKQEEVSAVVLQRAYRGHLARRGFICKK-TTSNKLENGGT   1930
NP_001070967.1    1880   SYEPITTTLRRKQEEVSAVVLQRAYRGHLARRGFICRK-ITSNKLENGGT   1928
NP_062139.2       1880   SYEPITTTLRRKQEEVSAVVLQRAYRGHLARRGFICRK-MASNKLENGGT   1928
XP_003643160.1    1880   SYEPITTTLRRKQEEVSAVVLQRAYRSRLARRGFISRR-PLPTKMENGGT   1928
NP_001038648.1    1857   SYEPITTTLRRKQEEVSAIVIQRSYRSHLASRGFICKRRPANNKLENGGG   1906
NP_571703.1       1851   SFEPITTTLRRKQEHMSAGVIQRAFRAHLIRKGFICKRLLSSSRLENGGT   1900
XP_004911965.1    1502   SYEPITTTLRRKQEDVSAVVIQQAYRRHLVKRGFISRK-RSTGKMENGGT   1550
```

NP_055006.1	1931	HREKKESTPSTASLPSYDSVTKPEKEKQQ--RAEEGRRERAKRQKEVRES	1978
XP_001141985.3	1865	HREKKESTPSTASLPSYDSVTKPEKEKQQ--RAEEGRRERAKRQKEVRES	1912
XP_001090295.1	1931	HREKKESTPSTASLPSYDSVTKPEKEKQQ--RAEEGRRERTKRQKEVRES	1978
XP_850134.1	1931	HREKKESTPSTASLPSYDSVTKPEKEKQQ--RAEEGRRERAKRQKEVRES	1978
NP_001180031.1	1931	HREKKESTPSTASLPSYDSVTKPEKEKQQ--RAEEGRRERAKRQKEVRES	1978
NP_001070967.1	1929	HREKKESTPSTASLPSYDSVTKPDKEKQQ--RAEEGRRERAKRQKEVRES	1976
NP_062139.2	1929	HRDKKESTPSTASLPSYDSVTKPDKEKQQ--RAEEGRRERAKRQKEVRES	1976
XP_003643160.1	1929	NREKKEGTPSTASLPSYDSVTKPEKEKQQ--RAEEGRRERAKRQKDVRES	1976
NP_001038648.1	1907	NQEKKEGTPSTASLPSYDSVTKPEKEKQDDNNEGKGRKEKGRNQKDVRES	1956
NP_571703.1	1901	NQDKKEGTPSTASLPSYDSVTKPEKEKLEE-SDSKGK--KGKNQKDVKES	1947
XP_004911965.1	1551	NMDKKEGTPSTASLPSYDSVTKPDKAKQE--RIEDAIRERAKKQKSVRES	1598

NP_055006.1	1979	KC	1980
XP_001141985.3	1913	KC	1914
XP_001090295.1	1979	KC	1980
XP_850134.1	1979	KC	1980
NP_001180031.1	1979	KC	1980
NP_001070967.1	1977	KC	1978
NP_062139.2	1977	KC	1978
XP_003643160.1	1977	KC	1978
NP_001038648.1	1957	KC	1958
NP_571703.1	1948	KF	1949
XP_004911965.1	1599	KC	1600
